# Supplementary material for: Association between Adherence to Swedish Dietary Guidelines and Mediterranean Diet and Risk of Stroke in a Swedish Population
Source: Nutrients. 2022 Mar 16;14(6):1253. doi: 10.3390/nu14061253 (PMC8954837; doi:10.3390/nu14061253)
Supplement: Supplementary file 1 [file nutrients-14-01253-s001.zip › nutrients-1639304-supplementary.pdf]

Supplementary materials

Table S1. Construction of the modified Mediterranean diet score.

Diet Component	Original Recommendation [1]	Serving Size	Recommendation in Servings/Week
**Encouraged**			
Fish and seafood	≥3 svg/wk	Fish: 125g<br>Seafood: 200g	≥3 svg/wk
Fruits and berries	≥3 units/day	100g [5]	≥21s vg/wk
Nuts and seeds	≥3 svg/wk	30g	≥3 svg/wk
Vegetables and legumes	Vegetables: ≥2 svg/d or ≥1 svg/d raw<br>Legumes: ≥3svg/wk	300g [4]	≥7 svg/wk
Vegetable oils [2]	≥ 4 tablespoons/d	13.5g [3]	≥28 svg/wk
Wine	≥7 svg/week	100g	≥7 svg/wk
**Discouraged**			
Butter, margarine, cream	<1 svg/d	12g	<7 svg/wk
Red and processed meat	<1 svg/d	125g	<7 svg/wk
Soda	<1 svg/d	200g	<7 svg/wk
Sweets and confectionary [6]	<3 svg/wk	Pastries: 50g<br>Sweets, chocolate: 30g	<3 svg/wk

[1] Original recommendations as stated by Martínez-González et al (ref). [2] Vegetable oils is a composite variable of vegetable oils to use in opposition of solid or animal fats for culinary purposes (the original MDS only considered olive oil for this item). [3] One tablespoon = 13.5 g according to Schröder et al 2011 (ref). [4] An average serving size of 300g was established for the pooled vegetable and legume intake to capture the differences I the recommendations established by the original mMDS. [5] The serving sizes for fruit and berry intake in the original MDS were calculated based on their respective carbohydrate content, however we established a standard serving size of 100 g to be used as an average portion size. [6] In the original MDS, this category refers only to commercial or store-bought baked goods, however we were unable to separate home-made from store-bought pastries in MDCS so both types are included in this category.
